# Biomechanical comparison of two surgical methods for Hallux Valgus deformity: Exploring the use of artificial neural networks as a decision-making tool for orthopedists

**DOI:** 10.1371/journal.pone.0297504

**Published:** 2024-02-13

**Authors:** Katarzyna Kaczmarczyk, Maria Zakynthinaki, Gabor Barton, Mateusz Baran, Andrzej Wit

**Affiliations:** 1 Faculty of Rehabilitation, Józef Piłsudski Academy of Physical Education, Warsaw, Poland; 2 School of Chemical and Environmental Engineering, Technical University of Crete, Chania, Greece; 3 Research Institute for Sport and Exercise Sciences, Liverpool John Moores University, Liverpool, United Kingdom; Ningbo University, CHINA

## Abstract

Hallux Valgus foot deformity affects gait performance. Common treatment options include distal oblique metatarsal osteotomy and chevron osteotomy. Nonetheless, the current process of selecting the appropriate osteotomy method poses potential biases and risks, due to its reliance on subjective human judgment and interpretation. The inherent variability among clinicians, the potential influence of individual clinical experiences, or inherent measurement limitations may contribute to inconsistent evaluations. To address this, incorporating objective tools like neural networks, renowned for effective classification and decision-making support, holds promise in identifying optimal surgical approaches. The objective of this cross-sectional study was twofold. Firstly, it aimed to investigate the feasibility of classifying patients based on the type of surgery. Secondly, it sought to explore the development of a decision-making tool to assist orthopedists in selecting the optimal surgical approach. To achieve this, gait parameters of twenty-three women with moderate to severe Hallux Valgus were analyzed. These patients underwent either distal oblique metatarsal osteotomy or chevron osteotomy. The parameters exhibiting differences in preoperative and postoperative values were identified through various statistical tests such as normalization, Shapiro-Wilk, non-parametric Wilcoxon, Student t, and paired difference tests. Two artificial neural networks were constructed for patient classification based on the type of surgery and to simulate an optimal surgery type considering postoperative walking speed. The results of the analysis demonstrated a strong correlation between surgery type and postoperative gait parameters, with the first neural network achieving a remarkable 100% accuracy in classification. Additionally, cases were identified where there was a mismatch with the surgeon’s decision. Our findings highlight the potential of artificial neural networks as a complementary tool for surgeons in making informed decisions. Addressing the study’s limitations, future research may investigate a wider range of orthopedic procedures, examine additional gait parameters and use more diverse and extensive datasets to enhance statistical robustness.

## Introduction

### Background

HALLUX Valgus (HV) is the most common foot deformity affecting approximately 30% of the population, causing pain and/or discomfort [1]. Although the exact cause is unknown, inherent, or acquired factors contribute to the condition’s development [2]. As HV progresses it impacts kinematic and kinetic parameters of gait due to continual pain, apprehension against loading the limb, and discomfort [3]. The potential consequences of leaving HV untreated can lead to various complications, including chronic pain, decreased mobility, and an increased risk of falls due to altered gait patterns. Furthermore, HV can progressively worsen, potentially necessitating more complex and invasive interventions in the future. Therefore, understanding the implications of untreated HV is crucial in making informed decisions about treatment strategies.

Although there are several conservative treatment options available [4–6], over 100 invasive correction techniques have been described [7], with no consensus on the most appropriate surgical technique [8–10]. For mild deformities, distal osteotomy, such as the distal chevron osteotomy, is usually applied, while Lapidus procedure and scarf osteotomy are also preferred by surgeons [11]. In recent years, minimally invasive surgery (MIS) has gained popularity as a treatment option for HV deformity. This approach is favored for its ability to minimize soft tissue damage, reduce surgical time, and enable quicker recovery when compared to traditional open surgeries [12]. Furthermore, recent studies [10, 13] have provided evidence suggesting that MIS procedures may be more effective than open surgeries in treating HV.

### Problem statement

The effectiveness of specific surgical procedures is generally evaluated by questionnaires and radiological examinations [14, 15]. However, in the case of HV, which is characterized by elevated plantar loading beneath the hallux and first metatarsal, as well as issues like reduced walking speed, shorter stride length, and an extended stance phase [16], it’s essential to emphasize the role of biomechanical assessments in both treatment and evaluation. Nonetheless, the existing literature lacks sufficient information for providing comprehensive insights into how different HV correction surgeries impact biomechanical gait parameters [13, 16–19]. Algorithms have been developed based on preoperative clinical and radiological features to categorize deformities and recommend the most suitable surgical procedure for individual patients [9]. While these algorithms offer valuable decision support, it is important to acknowledge their limitations. They generally rely on predefined criteria and data inputs, which may not encompass all individual patient considerations or account for subtleties in every clinical situation. To ensure personalized and optimal treatment decisions, it’s crucial to complement these algorithms with clinical data, static information from imaging modalities, and objective data derived from a patient’s dynamic movements.

Artificial neural networks (ANNs), on the other hand, are a versatile tool that has been used to diagnose several clinical conditions, including colon or colorectal cancer [20], multiple sclerosis [21], pancreatic disease [22], and early diabetes [23]. ANNs have made notable advancements within the field of orthopedic surgery. A recent review [24] explored their applications across five key orthopedic disciplines: joint reconstruction, spine, orthopedic oncology, trauma, and sports medicine. This review underscores the distinct advantages these algorithms bring in handling and interpreting intricate patient-specific data. Another recent review [25] delves into the potential of machine learning models to aid surgeons and enhance medical diagnoses and predictions within orthopedic surgery. This review highlights the remarkable achievements of artificial intelligence models in various tasks, such as fracture recognition, evaluating surgical suitability, and predicting surgical outcomes. A recent survey [26] underscores that the majority of current studies in this domain revolve around AI applications for imaging analysis, with a particular focus on fracture identification and classification. For a more comprehensive exploration of AI applications in foot and ankle surgery, interested readers are encouraged to consult [24–26] and the references cited therein.

While few studies [27–29] have shown ANNs to be useful in distinguishing gait patterns, none have attempted to categorize gait parameters in subjects after HV correction surgery or use ANNs as a decision-making tool in selecting the type of HV operation. ANNs offer numerous advantages over traditional algorithms, particularly in complex tasks like HV treatment decision-making. ANNs excel in capturing intricate nonlinear relationships among dynamic gate data, clinical and radiological features, and other factors crucial for understanding HV treatment decisions. They possess the capability to automatically extract relevant features from raw data, enhancing the precision of treatment recommendations based on a wide range of data types. ANNs demonstrate adaptability, continuously improving their decision-making accuracy as they learn from new patient data and evolving their internal parameters. Additionally, ANNs effectively handle the uncertainty and noise inherent in medical data, making robust predictions by learning patterns from diverse input sources. They seamlessly integrate information from various sources, which proves invaluable for HV treatment where decisions often rely on various types of qualitive and quantitative data. ANNs enable personalization, tailoring treatment recommendations to individual patient characteristics, an essential aspect of addressing each patient’s unique needs. These neural networks learn directly from data, making them suitable for situations where underlying relationships are complex or poorly understood, eliminating the need for manually crafted rules. Furthermore, they generalize well, generalizing insights from their training data to make predictions for new, unseen cases, an asset in the variable landscape of HV treatment.

Despite these advantages, it’s essential to acknowledge challenges such as the requirement for substantial training data, potential overfitting, and interpretability concerns. Nonetheless, in the context of complex tasks like HV treatment decision-making, ANNs’ ability to capture intricate relationships and provide personalized recommendations holds substantial promise, potentially serving as predictive tools to assess the likely impact of specific surgeries on a patient’s gait. This can greatly aid preoperative decision-making by allowing clinicians to inform patients about potential outcomes and helping patients establish realistic expectations.

### Methodology and objectives

This study focused on women with HV deformity who underwent two different surgical methods: distal oblique metatarsal osteotomy (DOM) and chevron osteotomy [1]. We utilized gait data from these patients, which included a range of spatiotemporal and foot pressure distribution parameters previously statistically analyzed in [1]. Building upon the findings from our previous research [1], which identified parameters distinguishing between the two HV groups before and after surgery, we employed statistical tests and data transformations for further investigation.

We developed two multi-layer perceptrons (MLP) in our analysis. The first MLP (ANN1) aimed to explore the feasibility of categorizing patients based on the type of surgery they received, considering changes in their biomechanical parameters before and after the procedure. Our second MLP, ANN2, was designed with the assumption that post-surgery walking speed could serve as a reliable indicator of the orthopedist’s decision quality regarding the type of operation. ANN2 was intended to function as a decision-making tool, assisting orthopedists in selecting the surgery type that would lead to greater improvements in a patient’s walking ability, thereby enhancing the overall effectiveness of the procedure.

The objectives of this study were:

- to determine the feasibility of classifying patients according to surgery type, based on their gait’s biomechanical parameters using ANNs, and
- to explore developing a decision-making tool using machine-learning techniques (ANNs) to assist orthopedists select the most appropriate surgical procedure.

## Materials and methods

### Participants

The present study utilized preoperative and postoperative gait performance data from twenty-three women with moderate to severe HV deformity in both feet who met the inclusion criteria of being between the ages of 40 and 70 with no other lower limb diseases. Participants with any other lower limb pathologies such as muscle weakness, foot-drop, or ischemic disease, previous surgeries like surgical fixation of foot or ankle fractures, or pain that could affect their gait, were excluded based on a medical interview. Seventy-six healthy women of the same age were included in the control group. The subjects were recruited continuously from 07/01/2020 to 30/06/2020. All subjects were informed about the study’s purpose and provided written informed consent. Approval of all ethical and experimental procedures and protocols was granted by the Józef Pilsudski University of Physical Education in Warsaw (SKE 01-33/2019) and performed in line with the Declaration of Helsinki. The experimental protocol and details of data collection can be found in [1].

Table 1 presents the demographic and anthropometric parameters of the participants. Those patients who underwent the most frequent surgery methods, DOM (n = 10) and chevron (n = 7), were qualified for further analysis [1].

**Table 1:** Anthropometric parameters, expressed as mean±SD.

Parameters	HV
	Group
	(n = 23)
Age [years]	55.49±6.48
Body mass [kg]	72.83±10.40
Body height [cm]	164.9±4.33
BMI [kg/m^2^]	26.71±3.06

### Parameters

Spatiotemporal and foot pressure distribution data was collected using the Zebris instrumented gait analysis system, provided by Zebris Medical GmbH in Tübingen, Germany. After two practice trials for familiarization, the participants completed three walking trials at their usual pace to mitigate any wayward effects during the initiation and termination of walking. The subjects performed these trials barefoot and unaided, starting and finishing each trial 2 meters before and after the mat to minimize acceleration and deceleration effects.

Twenty-two spatiotemporal parameters were recorded [1]: *left and right step length* (% of leg length), *left and right foot rotation* (degrees), *left and right step time* (s), *left and right stance phase* (% of gait cycle (GC)), *left and right loading respons*e (%GC), *left and right single support* (%GC), *left and right pre-swing* (%GC), *left and right swing phase* (%GC), *total double support* (%GC), *stride length* (% leg length), *stride time* (s), *step width* (cm), *cadence* (strides/min), *speed* (km/h). These spatiotemporal parameters offer crucial insights into motion and are directly relevant to clinical assessments and practical applications. Unlike more intricate kinematic and kinetic variables, these parameters are relatively simple to measure and calculate. They can be obtained using basic tools such as video analysis, motion capture systems, or even manual measurements. This accessibility makes them suitable for a wide range of research studies.

The foot pressure distribution parameters that analyze the course of the center of pressure (COP) during the selected step cycles were: *left and right gait line length* (mm), *left and right single support line* (mm), *ant/post position* (mm), *lateral symmetry* (mm):

- *Left and right gait line length* (mm) tracks the progression of the center of pressure (COP) for all recorded steps on one side of the body.
- *Left and right single support line* (mm) represents the average length of the lines depicting the COP’s progression while only that foot is in contact with the ground.
- *Ant/post position* (mm) illustrates the forward or backward shift of the COP intersection point in chronological order within the cyclogram display, accounting for all steps. The initial or zero position corresponds to the rearmost point where the heel initially makes contact with the ground.
- *Lateral symmetry* (mm) depicts the left/right shift of the COP intersection point in chronological order within the cyclogram display, considering all steps. A negative value indicates a shift to the left, while a positive value indicates a shift to the right.

### Statistical analysis and artificial neural networks

The recorded data were expressed as mean ± standard deviation and analysed by use of STATISTICA version 13.3, PL.iso, TIBCO Software Inc., Palo Alto, California, USA. To further investigate recent findings [1], regarding the identification of parameters that distinguish between the two HV groups before and after surgery, we utilized statistical tests and transformations on the data, as described below. To address the limited statistical power of the analysis, we employed the paired difference (delta) test.

To eliminate the influence of baseline values, when evaluating the differences between preoperative and postoperative test, all parameters were normalized:

$$u_{i}=\frac{x_{i}-x_{mean}}{SD}$$

where *u*_*i*_ is the normalized value of the given parameter (z-score), *x*_*mean*_ is the mean value of this parameter in the control group, and *SD* is its standard deviation in the control group.

The normalized *u*_*i*_ values were further transformed into score values as per the T scale:

$$T_{i}=u_{i}*10+50$$

where *T*_*i*_ is the score value on *T* scale.

The Shapiro-Wilk test showed that most of the studied parameters did not show a normal distribution and so the non-parametric Wilcoxon test for repeated trials was (*p<0*.*05*). We verified the results of the Wilcoxon test by examining the differences (deltas) between the results of the repeated tests in each patient:

$$\Delta_{abs}=\sqrt{\frac{\sum {\left(D_{i}-D_{avg}\right)}^{2}}{2(n-1)}}$$

where *Δ*_*abs*_ is the absolute value of the difference between the values of the specified parameter “before” and “after” the procedure in the whole group of patients, *D*_*i*_ is the difference in the values of a single patient and *D*_*avg*_ is the average value of the differences of all participants.

The percentage difference between the test repetitions in each patient was calculated as:

$$\Delta_{\%}=\frac{\Delta_{abs}}{\frac{x_{1}+x_{2}}{2}}*100$$

where *Δ*_*%*_ is the difference of the specified parameters “before” and “after” the procedure in the whole group of patients in percentage, *x*_*1*_ is the mean value of the specified parameter “before”, and *x*_*2*_ is the mean value of the specified parameter “after” the procedure in the whole group of patients. The result of percentage difference was evaluated according to the formula

$$t=\frac{{SE}_{\Delta }}{x_{1}-x_{2}}$$

where *t* is Student test for paired samples and *SE*_*Δ*_ is the standard error of the mean difference (D_1_).

The dispersion of the results in the group of subjects for each parameter was estimated as follows:

$$\%SD=\frac{\pm SD}{x_{mean}}*100$$

The following criteria were used to assess the variability of the results around the mean: <5%—slight, 6–10% moderate, 11–20% significant, 21–50% high, >50% very high.

The nonparametric Wilcoxon matched-pairs signed-rank test was used to check if there were differences in the two scores that were being compared. The effect size for the Wilcoxon test was determined by use of G*Power v.3.1.9.7. The effect sizes were assessed using the following criteria: <0.2—trivial, 0.2–0.6—small, 0.6–1.2, moderate, 1.2–2.0—large, and >2.0—very large.

Two multi-layer perceptrons (MLP) were constructed for prediction and simulation. The first (ANN1) was employed to examine the feasibility of categorizing patients according to the type of surgery, based on the alteration in their biomechanical parameters before and after surgery. ANN1 was fed with the parameters that showed statistically significant differences between the preoperative and postoperative values (*p<0*.*05*), and built with 1 hidden layer with 11 neurons, and one qualitative double output (operation type). It was trained by use of the second-order optimization algorithm Broyden–Fletcher–Goldfarb–Shanno (BFGS), SOS error function, and a linear activation for the hidden and output layers.

ANN2 was constructed based on the assumption that the walking speed after surgery is a reliable indicator of the quality of the orthopedist’s decision regarding the type of operation performed. Its aim was to act as a decision-making tool, enabling the orthopaedist to choose the operation type that improves the patient’s walking to a greater extent, thereby enhancing the overall effectiveness of the procedure. The input to ANN2 was again the parameters that showed statistically significant differences in their preoperative and postoperative values (*p<0*.*05*), plus one categorical input parameter, the surgery type (chevron or DOM). ANN2 was built with 1 hidden layer with 15 neurons and 1 output (the after-surgery speed). It was trained by use of the same algorithms as ANN1, however exponential and logistic function activation were applied for the hidden and the output layers respectively.

During the process of constructing the two ANNs, the complete dataset was partitioned into three distinct subsets: training (40%), testing (30%), and validation (30%, utilized for fine-tuning the model’s parameters and preventing overfitting). It’s important to emphasize that the selection of individuals for these specific subgroups was performed through a random process, ensuring a representative and unbiased distribution of subjects across the three subsets.

## Results

### Examining the differences of the spatiotemporal parameters preoperative and postoperative

Table 2 complements the statistical findings presented in the study of [1].

**Table 2:** Differences in gait parameters before and after surgery, as calculated by Wilcoxon (Z) and Student-t tests.

Parameter/Operation type	Z	p	%Delta	t	p
*Step length*, *% of leg length*, *right*					
Both operations	2.81	0.005**	20.2	3.71	0.01
DOM	2.08	0.037*	0.3	2.77	0.02
Chevron	1.86	0.062	23,0		0.1
*Foot rotation (°)*, *right*					
Both operations	3.01	0.003**	14.2	3.95	0.001
DOM	2.40	0.017*	9.7	-3.59	0.01
Chevron	2.03	0.042*	18.5	-2.38	0.05
*Step time (s)*, *right*					
Both operations	1.91	0.059	16.6	-1.83	0.1
DOM	1.83	0.066	19.6	-1.84	0.1
Chevron	0.34	0,735	10.9	-0.40	Ns
*Stance phase (%GC)*, *right*					
Both operations	2.05	0.039*	17.7	-2.42	0.05
DOM	1.68	0.092	22.9	-1.93	0.1
Chevron	1.52	0.128	9.8	-1.51	Ns
*Loading response (%GC)*, *right*					
Both operations	1.87	0.061	13.5	-1.92	0.1
DOM	1.37	0.169	15.3	-1.57	Ns
Chevron	1.35	0.176	11.5	-1,31	Ns
*Single support (%GC)*, *right*					
Both operations	1.73	0.084	32.6	2.02	0.1
DOM	1.68	0.093	36.4	1.97	0.1
Chevron	0.676	0.499	26.1	0.65	Ns
*Pre-swing (%GC)*, *right*					
Both operations	1.68	0.093	15.9	-2.09	0.1
DOM	1.78	0.074	19.0	-2.13	0.1
Chevron	0.17	0.866	10.2	-0.531	Ns
*Swing phase (%GC)*, *right*					
Both operations	2.06	0.039*	21.7	2.43	0.05
DOM	1.68	0.092	24.0	1.93	0.1
Chevron	1.52	0.128	13.6	1.68	Ns
*Total double support (%GC)*					
Both operations	1.82	0.068	1.8	-2.17	0.02
DOM	1.68	0.093	2,1	-1.87	0.1
Chevron	1.01	0.311	1.0	-1.23	Ns
*Stride length*, *% of leg length*					
Both operations	3.62	0.001**	30.0	4.14	0.001
DOM	2.80	0.005**	32.9	9.85	0.001
Chevron	2.37	0.018*	27.6	2.62	0.05
*Stride time (s)*					
Both operations	2.20	0.028*	18.4	-1.92	0.01
DOM	1.89	0.059	23.6	-1.67	Ns
Chevron	1.18	0.237	10.5	-0.96	Ns
*Step width (cm)*					
Both operations	1.40	0.163	9.7	-1.51	Ns
DOM	0.36	0.721	8.8	-0.26	Ns
Chevron	1.69	0.091	10.1	-1.98	0.1
*Cadence (strides/min)*					
Both operations	2.15	0.031*	22.4	2.06	0.05
DOM	1.88	0.059	24.8	1.84	0.1
Chevron	1.18	0.237	16.5	0.91	Ns
*Speed (km/h)*					
Both operations	2.63	0.009**	27.3	3.55	0.01
DOM	1.99	0.047*	28.9	2.74	0.05
Chevron	1.69	0.091	25.5	1.94	0.1
*Gait line length (mm)*, *right*					
Both operations	0.31	0.758	13.6	-0.63	Ns
DOM	1.36	0.169	13.4	-1.70	Ns
Chevron	1.18	0.237	11.15	1.19	Ns
*Single support line (mm)*, *right*					
Both operations	0.07	0.943	10.0	-0.01	Ns
DOM	0.25	0.799	9.4	-0.20	Ns
Chevron	0.34	0.735	11.6	0.19	Ns

Several spatiotemporal parameters can be observed to exhibit significant differences (*p<0*.*05*) between preoperative and postoperative gait performance: Foot *rotation* and *stride length as a percentage of leg length* exhibit significant differences (*p<0*.*05*) for both types of surgeries, as well as for each individual surgery type. Two parameters (*step length*, *% of leg length*, *right* and *speed*) for one surgery type and four parameters for the overall group (*stance phase (%GC)*, *right*, *swing phase (GC%)*, *right*, *stride time* and *cadence*), also demonstrate significant differences (*p<0*.*05*). The paired difference test produced identical results for both methods, differing only in the significance level, due to the different calculation algorithms.

### Classifying the patients by type of surgery using ANN

The input parameters to ANN1 were the ones that showed statistically significant differences (Table 2, *p<0*.*05*), excluding the parameter *foot rotation*, which was omitted, due to high measurement uncertainty (in the experimental group the value of this parameter is $\bar{x}$ = 4.78 ± 4.91deg, and in the control group $\bar{x}$ = 4.62 ± 4.56deg). For each parameter, we calculated a normalized preoperative value and determined the percentage of improvement between the preoperative and postoperative values. Thus, ANN1 consisted of 14 quantitative input cells (taken from the parameters shown in Table 2, only for the right limb) and one qualitative double output (comprising two continuous output nodes representing predictions for surgery type). Statistical analysis, including Wilcoxon Z and Student t-tests, indicated no significant differences in the parameters of the ANNs between the three subgroups (p>0.08). This finding underscores the representativeness of the three subgroups within our dataset.

The output of ANN1, which predicts the surgery type, is presented in Table 3. As the output nodes represent distinct categories, the node with the highest value was selected as the predicted category. The outcomes presented demonstrate that ANN1 correctly classified individual patients according to their medical histories, affirming the proposition that a strong association exists between the type of surgery performed and the descriptive gait parameters before and after surgery (difference expressed on T scale).

**Table 3:** Actual and predicted patient classification according to surgery type.

Actual surgery type	DOM Predicted	Chevron Predicted	% Correct
DOM	10	0	100
Chevron	0	7	100
	*p = 0*.*5882*	*p = 0*.*4118*	

The threshold for categorizing each output node was set at 0.5. Notably, a range of thresholds (from 0.3 to 0.6) was used to compute diverse metrics, including Accuracy, Precision, Recall, F1 Score, AUC-PR, and MCC. Remarkably, the model consistently attains 100% accuracy, with all metrics consistently registering a perfect score of 1.00. The confusion matrix underscores this exceptional performance, revealing zero false positives and false negatives, along with 100% true positives and true negatives. However, while the numbers suggest perfection, it’s essential to exercise caution when interpreting such results, especially when dealing with a small dataset.

### Predicting the optimal surgical operation type using ANN

ANN2 was built having as input the preoperative values of the 7 gait parameters, which showed statistically significant differences in their preoperative and postoperative values (*p<0*.*05*), plus one categorical input parameter, the surgery type (chevron or DOM). The parameter *foot rotation* was omitted as before. The output of ANN2 was the after-surgery "normalized speed". The network reflects the actual data well (*r = 0*.*9957*).

Table 4 shows the after-surgery walking speeds, expressed in terms of percentage difference between normalized pre-and post-intervention values, as resulted from the performed surgery types (real data) and the alternative surgery type (simulated by ANN2). The last column of Table 4 indicates the match between the orthopedist’ decision and the neural network’s simulation in terms of true (T) and false (F).

**Table 4:** Effectiveness of the performed and the simulated surgery types, in relation to post-intervention walking speed.

Performed surgery type	Measured speed (% normalized)	Simulated surgery type	Simulated speed (% normalized)	Match
1.DOM	73.4	CHEVRON	95.1	F
2.CHEVRON	84.4	DOM	54.2	T
3.DOM	59.5	CHEVRON	91.9	F
4.DOM	85.8	CHEVRON	95.2	F
5.DOM	87.1	CHEVRON	95.8	F
6.DOM	76.7	CHEVRON	95.8	F
7.DOM	99.3	CHEVRON	53.5	T
8.CHEVRON	79.5	DOM	63.1	T
9.DOM	61.1	CHEVRON	85.1	F
10.CHEVRON	79.5	DOM	87.2	F
11.CHEVRON	86.5	DOM	77.6	T
12.CHEVRON	56.3	DOM	35.1	T
13.DOM	92.7	CHEVRON	63.1	T
14.DOM	81.2	CHEVRON	93.4	F
15.CHEVRON	74.4	DOM	58.8	T
16.CHEVRON	95.8	DOM	50.1	T
17. DOM	101.4	CHEVRON	93.8	T

The differences between the two sets are overall negligible at five percentage points. In the Wilcoxon test, the values found were, *z = 0*.*6153*, *p <* .*5382*, *r*_*c*_ *= -0*.*0130*. A low r_c_ value and a high value of the dispersion index indicate a large interindividual variation in the group: A low r_c_ and a high value of the dispersion index (%SD_measured_ = 16.4%, %SD_simulated_ = 26.7%) indicate a large inter-individual variation in the group. The simulation results indicate, however, that in the case of DOM surgery, the neural network simulation did not match the surgeon’s choice in almost 2/3 (70%) of the patients, and in the case of chevron surgery in 1 patient (15%).

## Discussion

Surgical interventions have been reported to alter the biomechanics of the foot [30], disrupting weight transfer and power generation in the push-off phase of gait, with the specific effects varying, depending on the type of surgery performed. Studies have reported longer flat-foot time after modified Lapidus procedure [14, 31, 32], slower walking speed following scarf osteotomies, and increase in step time and decreased walking speed in the operated limb, respectively. Shorter single support lines in patients that underwent chevron and DOM techniques have been reported [1].

Several studies have compared chevron osteotomy with other distal metatarsal osteotomies [33], however the effectiveness of the specific surgical procedures has, in most cases, been evaluated by questionnaires, scales and radiological examination. In the present study we examined the differences between the preoperative and postoperative values of gait parameters of patients who underwent DOM and chevron osteotomies and used an ANN to classify the patients based on the type of surgery. We found a close relationship between surgery type and gait parameters after surgery: the results showed that the two groups are distinctively different and fully separable, with 100% classification accuracy. This remarkable precision is noteworthy, especially given the constraints of our small dataset and can be attributed to several factors. Firstly, the homogeneity of the datasets is evident, as the absence of statistically significant differences between the training, testing, and validation datasets (p>0.08) suggests shared characteristics among the data subsets. Additionally, the features employed for classification seem to exhibit a high degree of separability between the two surgery types, facilitating the ANN1’s capacity to accurately differentiate and classify cases. However, only two parameters out of 22 spatiotemporal parameters of gait (*foot rotation* and *stride length*) showed significant differences between the preoperative and postoperative values (*p<0*.*05*). In the literature, the great potential of ANNs in distinguishing gait patterns has been reported in various studies with accuracy 83.3% [27, 34], to 95% [28]. A study examining the complete progression of lower limb joint angle changes during the gait cycle in post-stroke patients found success rates ranging from 100% for the knee joint to 86% for the frontal motion of the hip joint [29].

The present study underscores the significance of specific gait parameters in evaluating the effectiveness of orthopedic surgeries. By identifying and closely monitoring these parameters before and after surgery, clinicians can enhance their ability to customize treatment plans and rehabilitation strategies, ultimately optimizing patient recovery. The strong correlation we’ve observed between the type of orthopedic surgery and post-intervention gait parameters highlights the potential for tailoring surgical approaches to individual patient needs. Different surgical techniques can have varying effects on a patient’s gait, and a deeper understanding of these distinctions can aid surgeons in selecting the most suitable procedure for each patient. This, in turn, holds the promise of improving the overall success of surgeries and expediting recovery.

ANNs have also been reported to serve as decision-making tools, in various fields, due to their ability to model complex and nonlinear relationships. Such usage of ANNs extends in numerous applications, including engineering [35], statistics and stock market [36], medicine [37], and exercise physiology [38], among others. Predictions based on ANNs are more accurate than regression models, thanks to the hidden layers that filter out redundant information. In orthopedic knee surgery, ANNs have been reported as an aid in determining whether a patient was a candidate for surgery [25]. Referring to knee arthroplasty, the authors in the same review discuss the role of ANNs as an adviser to the type of this specific operation. There are no studies on using ANN as a decision-making tool to aid in selecting the most suitable type of surgery for HV patients.

The process of surgical decision-making is crucial in planning for surgery and can greatly impact the outcome of the surgical intervention. Walking speed after surgery is a common metric used to evaluate the effectiveness of the procedure and assess the patient’s postoperative condition, providing valuable insights into their mobility and functional ability. This study assessed the z-score of post-surgery walking speed and compared actual values from the performed operation to hypothetical values generated by ANN simulation for an alternative operation type. A limitation of this approach is that the trained ANN2 was expected to extrapolate to preoperative gait parameters combined with the opposite type of operation as input, even though such data were not presented during training. It is interesting to note that, as the study showed, in the case of DOM intervention, almost 2/3 (70%) of the patients were assigned a simulated optimal surgery type different from the surgeon’s decision; a chevron surgery would have resulted to greater post-surgery walking speed values. In the case of chevron surgery, the surgeon’s decision showed a mismatch with the neural network’s simulation in only one patient.

Due to testing the responses of the trained ANN2 with input patterns not covered during training, these results should merely be treated as a novel and experimental use of neural networks. With further efforts to avoid extrapolation, which is known to lead to unpredictable results, this approach could lead to development of a complementary tool to determine the best surgical approach and thus help surgeons make informed decisions. Of course, we should note that our suggested ANN can only act as support to clinical decision making; it can serve as an adviser to the orthopedist and not as a replacement of their expertise and experience. The final decision as to the type of surgery should always rely on the experienced judgment of the orthopedic surgeon.

We acknowledge several limitations in our study. Firstly, our data was derived from a limited sample size and collected trials, primarily due to the restricted availability of participants who underwent either chevron or distal metatarsal osteotomy (DOM) interventions. We must also consider that factors like participants’ familiarity with the testing environment or the presence of an observer might have influenced gait behavior, potentially introducing bias. Splitting the data into training, testing, and validation subsets is crucial for evaluating the models’ performance. However, due to our small sample size, each subset may not fully represent the data distribution, possibly leading to bias in model assessment.

Regarding ANNs, it’s important to acknowledge their inherent limitation–the inability to extrapolate. Additionally, achieving remarkable accuracy with our approach raises concerns about potential overfitting, given that hyperparameter tuning was not performed. Furthermore, the choice of ANN architecture could impact the model’s complexity and its ability to generalize to new data.

In the realm of clinical implementation, several practical challenges may arise when integrating the proposed approach into orthopedic practice. One significant hurdle lies in the need for consistent and standardized data collection procedures. Achieving reliable and comparable gait parameter measurements across different clinical settings is crucial for the model’s accuracy and generalizability. Variances in equipment, testing environments, or personnel conducting the assessments could introduce inconsistencies, potentially impacting the reliability of the predictions. Furthermore, the successful application of the ANN as a decision-support tool hinges on its adaptability to the diverse patient population encountered in clinical practice. Patient-specific factors, such as comorbidities, unique anatomical variations, or distinct rehabilitation needs, may pose challenges to the model’s ability to provide universally applicable recommendations. Moreover, the implementation of any new technology in a clinical setting necessitates thorough training and familiarization among healthcare professionals. Orthopedic surgeons and clinical staff would need to acquire the necessary skills to interpret and integrate the ANN-generated insights into their decision-making process effectively. This requires time, resources, and ongoing support to ensure a smooth transition and sustained use in everyday clinical practice. Despite these challenges, recognizing the potential benefits of the ANN as a complementary tool in surgical decision-making underscores the importance of overcoming these practical obstacles. With meticulous attention to standardization, adaptability, and comprehensive training, the integration of such technologies holds promise in enhancing personalized patient care and optimizing surgical outcomes within the orthopedic field.

While our results offer valuable insights based on the available data and methodology, it’s crucial to remain vigilant and continue refining our approach. Future research endeavours should aim to build upon the foundational aspects of this study, addressing the inherent limitations and broadening the investigative scope for a more comprehensive understanding of the intricate relationship between orthopedic surgeries and gait parameters. Potential directions for future research include:

- Enlarging sample sizes to enhance statistical robustness.
- Incorporating more diverse populations to ensure broader applicability.
- Exploring additional gait parameters or refining existing ones for a nuanced assessment.
- Incorporating additional clinical outcome measures beyond gait parameters, such as pain levels, patient satisfaction, or functional scores.
- Integrating patient-reported outcomes and perspectives to capture subjective experiences.
- Comparing the performance of the ANNs with traditional methods of assessing gait outcomes, such as questionnaires, scales, and radiological examinations.
- Investigating the practical challenges associated with the clinical implementation of the ANN.
- Conducting a comprehensive cost-benefit analysis to evaluate economic implications.
- Exploring various types of orthopedic operations to understand their distinct effects on gait.
- Extending the application of the study to different pathologies within orthopedics.

These proposed directions aim to advance our understanding and contribute to the ongoing refinement of methodologies in orthopedic research.

## Conclusions

This study utilized preoperative and postoperative gait parameter values of patients who underwent chevron and DOM surgeries for Hallux Valgus treatment. Based on the statistical analysis, only two of the recorded gait parameters exhibited significant differences between their preoperative and postoperative values for both types of operation, with additional parameters exhibiting significant differences when considering each operation type separately (*p<0*.*05*). The statistical results indicated a strong correlation between the type of orthopaedic surgery and postoperative gait parameters, and the ANN achieved classification accuracy of 100%. The study also introduced a second ANN demonstrating the potential of developing a complementary tool to determine the best surgical approach.

Our study’s findings hold promise for advancing orthopedic surgery. Researchers can explore various types of operations, work with larger datasets, incorporate more diverse populations or additional gait parameters, or refine existing ones to enhance the predictive accuracy of ANNs. This endeavor promises continuous improvement in surgical techniques and patient care.

## Supporting information

S1 Checklist STROBE statement—Checklist of items that should be included in reports of observational studies. (DOCX)

S1 Data Data set underlying the results of this study. (XLSX)
