# Supplementary material for: Biomechanical comparison of two surgical methods for Hallux Valgus deformity: Exploring the use of artificial neural networks as a decision-making tool for orthopedists
Source: PLoS One. 2024 Feb 13;19(2):e0297504. doi: 10.1371/journal.pone.0297504 (PMC10863859; doi:10.1371/journal.pone.0297504)
Supplement: S1 Checklist — (DOCX) [file pone.0297504.s001.docx]

STROBE Statement—checklist of items that should be included in reports of observational studies

|  | | | Item No. | Recommendation | | Page  No. | | | Relevant text from manuscript |
| --- | --- | --- | --- | --- | --- | --- | --- | --- | --- |
| **Title and abstract** | | | 1 | (*a*) Indicate the study’s design with a commonly used term in the title or the abstract | | 2 | | | "Abstact: The objective of this cross-sectional study aimed..."  Line 22 |
|  |  |  |  | (*b*) Provide in the abstract an informative and balanced summary of what was done and what was found | | 2 | | | From " To achieve this, gait parameters of twenty-three women " ... to end of Abstract.  Lines 25-36 |
| Introduction | | | | | | | | |  |
| Background/rationale | | | 2 | Explain the scientific background and rationale for the investigation being reported | | 3 | | | From " Hallux Valgus (HV) is the most common foot deformity"... to " use ANNs as a decision-making tool in selecting the type of HV operation."  Lines 42-60 |
| Objectives | | | 3 | State specific objectives, including any prespecified hypotheses | | 3 | | | From "This study was based on gait data"... to "to assist orthopedists select the most appropriate surgical procedure."  Lines 61-65 |
| Methods | | | | | | | | |  |
| Study design | | | 4 | Present key elements of study design early in the paper | | 4-7 | | | " The present study utilized preoperative and postoperative gait performance data from twenty-three women with moderate to severe HV deformity in both feet who met the inclusion criteria" Lines 68-70  "Twenty-two spatiotemporal parameters were recorded"  Line 86  Statistical Analysis section, line 90 thereafter.  " Two multi-layer perceptrons (MLP) were constructed for prediction and simulation."  Line 130 |
| Setting | | | 5 | Describe the setting, locations, and relevant dates, including periods of recruitment, exposure, follow-up, and data collection | | 4 | | | "the experimental protocol and details of data collection can be found in [1]”.  Line 77 |
| Participants | | | 6 | (*a*) *Cohort study*—Give the eligibility criteria, and the sources and methods of selection of participants. Describe methods of follow-up  *Case-control study*—Give the eligibility criteria, and the sources and methods of case ascertainment and control selection. Give the rationale for the choice of cases and controls  *Cross-sectional study*—Give the eligibility criteria, and the sources and methods of selection of participants | | 4 | | | "The present study utilized preoperative and postoperative gait performance” … to “included in the control group."  Lines 68-73  "Those patients who underwent the most frequent surgery methods, DOM (n=7) and chevron (n=10), were qualified for further analysis."  Lines 79-81 |
|  |  |  |  | (*b*)*Cohort study*—For matched studies, give matching criteria and number of exposed and unexposed  *Case-control study*—For matched studies, give matching criteria and the number of controls per case | |  | | |  |
| Variables | | | 7 | Clearly define all outcomes, exposures, predictors, potential confounders, and effect modifiers. Give diagnostic criteria, if applicable | | 4-7 | | | "With the numbers available, no significant differences with respect to age, body mass, height and BMI were detected between the experimental and the control groups (*p>.08*)."  Lines 78-79  "Twenty-two spatiotemporal parameters were recorded"  Line 83  " The recorded data were expressed as mean ± standard deviation and analysed by use of STATISTICA"  Lines 91,92  "To address the limited statistical power of the analysis, we employed the paired difference (delta) test." Lines 95,96  "To eliminate the influence of baseline values, when evaluating the differences between preoperative and postoperative test, all parameters were normalized"  Lines 97-98  "The normalized values were further transformed into score values as per the T scale"  Line 12  "The Shapiro-Wilk test showed that most of the studied parameters did not show a normal distribution and so the non-parametric Wilcoxon test for repeated trials was (*p<.05*). We verified the results of the Wilcoxon test by examining the differences (deltas) between the results of the repeated tests in each patient"  Lines 105-108  "Two multi-layer perceptrons (MLP) were constructed “… to “and validation (30%)."  Lines 130-147 |
| Data sources/measurement | | | 8* | For each variable of interest, give sources of data and details of methods of assessment (measurement). Describe comparability of assessment methods if there is more than one group | | 4 | | | "the experimental protocol and details of data collection can be found in [1]”.  Line 77 |
| Bias | | | 9 | Describe any efforts to address potential sources of bias | | 5 | | | Shapiro-Wilk test, line 105  non-parametric Wilcoxon test, line 106  differences (deltas) between the results of the repeated tests, line 107 |
| Study size | | | 10 | Explain how the study size was arrived at | | 4 | | | "Those patients who underwent the most frequent surgery methods, DOM (n=7) and chevron (n=10), were qualified for further analysis."  Lines 79-81 |
| Quantitative variables | | 11 | | Explain how quantitative variables were handled in the analyses. If applicable, describe which groupings were chosen and why | | 3-6 | | Section "Statistical Analysis"  Lines 90-28 | |
| Statistical methods | | 12 | | (*a*) Describe all statistical methods, including those used to control for confounding | | 3-6 | | Section "Statistical Analysis"  Lines 90-128 | |
|  |  |  |  | (*b*) Describe any methods used to examine subgroups and interactions | | 6-7 | | Section “Artificial Neural Networks”  Lines 129-147 | |
|  |  |  |  | (*c*) Explain how missing data were addressed | |  | | N/A | |
|  |  |  |  | (*d*) *Cohort study*—If applicable, explain how loss to follow-up was addressed  *Case-control study*—If applicable, explain how matching of cases and controls was addressed  *Cross-sectional study*—If applicable, describe analytical methods taking account of sampling strategy | | 4-7 | |  | |
|  |  |  |  | (*e*) Describe any sensitivity analyses | | 6-7 | | Section "Statistical Analysis"  Lines 90-128 | |
| Results | | | | | | | | | |
| Participants | | 13* | | (a) Report numbers of individuals at each stage of study—eg numbers potentially eligible, examined for eligibility, confirmed eligible, included in the study, completing follow-up, and analysed | | 4 | | All information regarding the participants can be found in Kaczmarczyk et al. (2021).  "the experimental protocol and details of data collection can be found in [1]”. Line 77 | |
|  |  |  |  | (b) Give reasons for non-participation at each stage | |  | | Same as above | |
|  |  |  |  | (c) Consider use of a flow diagram | |  | | Same as above | |
| Descriptive data | | 14* | | (a) Give characteristics of study participants (eg demographic, clinical, social) and information on exposures and potential confounders | |  | | Same as above | |
|  |  |  |  | (b) Indicate number of participants with missing data for each variable of interest | |  | | Same as above | |
|  |  |  |  | (c) *Cohort study*—Summarise follow-up time (eg, average and total amount) | |  | | Same as above | |
| Outcome data | | 15* | | *Cohort study*—Report numbers of outcome events or summary measures over time | |  | | Same as above | |
|  |  |  |  | *Case-control study—*Report numbers in each exposure category, or summary measures of exposure | |  | | Same as above | |
|  |  |  |  | *Cross-sectional study—*Report numbers of outcome events or summary measures | |  | | Same as above | |
| Main results | | 16 | | (*a*) Give unadjusted estimates and, if applicable, confounder-adjusted estimates and their precision (eg, 95% confidence interval). Make clear which confounders were adjusted for and why they were included | | 10 | | “Foot *rotation* and *stride length as a percentage of leg length* exhibit significant differences (*p<.05*) for both types of surgeries, as well as for each individual surgery type. Two parameters (*step length, % of leg length, right* and *speed*) for one surgery type and four parameters for the overall group (*stance phase (%GC), right*, *swing phase (GC%), right*, *stride time* and *cadence*), also demonstrate significant differences (*p<.05*). The paired difference test produced identical results for both methods, differing only in the significance level, due to the different calculation algorithms. “  Lines 158-164 | |
|  |  |  |  | (*b*) Report category boundaries when continuous variables were categorized | |  | |  | |
|  |  |  |  | (*c*) If relevant, consider translating estimates of relative risk into absolute risk for a meaningful time period | |  | |  | |
| Other analyses | 17 | | Report other analyses done—eg analyses of subgroups and interactions, and sensitivity analyses | | 11-13 | | “The outcomes presented demonstrate that ANN1 correctly classified individual patients according to their medical histories, affirming the proposition that a strong association exists between the type of surgery performed and the descriptive gait parameters before and after surgery (difference expressed on T scale).”  Lines 175-178  ANN2: “The simulation results indicate, however, that in the case of DOM surgery, the neural network simulation did not match the surgeon’s choice in almost 2/3 (70%) of the patients, and in the case of chevron surgery in 1 (15%) patient.”  Lines 200-202 | | |
| Discussion | | | | | | | | | |
| Key results | 18 | | Summarise key results with reference to study objectives | | 15 | | Conclusions section, Lines 253-263 | | |
| Limitations | 19 | | Discuss limitations of the study, taking into account sources of potential bias or imprecision. Discuss both direction and magnitude of any potential bias | | 15 | | “Limitations of the present study are the small sample size due to the limited availability of participants undergoing only chevron or DOM intervention, and the inherent inability of neural networks to extrapolate. “  Lines 260-262 | | |
| Interpretation | 20 | | Give a cautious overall interpretation of results considering objectives, limitations, multiplicity of analyses, results from similar studies, and other relevant evidence | | 15 | | From “Due to testing the responses of the trained ANN2“… to “the experienced judgment of the orthopedic surgeon..”  Lines 244-251 | | |
| Generalisability | 21 | | Discuss the generalisability (external validity) of the study results | |  | | See above | | |
| Other information | | |  | | | | | | |
| Funding | 22 | | Give the source of funding and the role of the funders for the present study and, if applicable, for the original study on which the present article is based | | Title page | | “The Józef Pilsudski University of Physical Education in Warsaw, Research Group no. 3 received support from the Polish Ministry of Science and Higher Education during the period of 2020-2022. The support was granted for the purpose of carrying out research on "Motor system diagnostics in selected dysfunctions as a basis for planning the rehabilitation process." | | |

*Give information separately for cases and controls in case-control studies and, if applicable, for exposed and unexposed groups in cohort and cross-sectional studies.

**Note:** An Explanation and Elaboration article discusses each checklist item and gives methodological background and published examples of transparent reporting. The STROBE checklist is best used in conjunction with this article (freely available on the Web sites of PLoS Medicine at http://www.plosmedicine.org/, Annals of Internal Medicine at http://www.annals.org/, and Epidemiology at http://www.epidem.com/). Information on the STROBE Initiative is available at www.strobe-statement.org.
